# 3D printing technology will eventually eliminate the need of purchasing commercial phantoms for clinical medical physics QA procedures

**DOI:** 10.1002/acm2.12392

**Published:** 2018-06-26

**Authors:** Eric Ehler, Daniel Craft, Yi Rong

**Affiliations:** ^1^ Department of Radiation Oncology University of Minnesota Minneapolis MN 55455 USA; ^2^ Department of Radiation Physics University of Texas MD Anderson Cancer Center Houston TX 77030 USA; ^3^ Department of Radiation Oncology University of California Davis Comprehensive Cancer Center Sacramento CA 95817 USA

## 1 INTRODUCTION

3D printing is not a new concept. The recent advances in printing speed, technology, and material selection are promoting its significant impacts in several industries, including health care. For our medical physics field, researchers are also finding its applications in various clinical aspects. However, the interests still remain in a few academic centers who have the luxuries of owning such an unconventional device in the radiation oncology department, or collaborating with a local 3D printing lab. As the 3D printing technology is becoming an unstoppable driving force in manufacturing revolution, are we also envisioning a future that 3D printing will become as common as a block‐cutting machine in a radiation oncology department? In this debate, we invited two researchers who are experienced in studying the clinical use of 3D printing in medical physics field. Dr. Eric Ehler is arguing for the proposition that “*3D printing technology will eventually eliminate the need of purchasing commercial phantoms for clinical medical physics QA procedures*” and Dr. Daniel Craft is arguing against.

Dr. Eric Ehler is an Assistant Professor in the Department of Radiation Oncology at the University of Minnesota. He is the medical physics residency program director at the University of Minnesota Medical Center. His education and research interests are 3D printing, pediatric radiotherapy, radiation dosimetry, and machine learning.

Dr. Daniel Craft is currently a medical physics resident at The Mayo Clinic in Phoenix, AZ. Prior to the beginning of his residency, Dr. Craft was a graduate research assistant and PhD student at the University of Texas MD Anderson Cancer Center in Houston Texas, where he studied techniques to deliver postmastectomy radiation therapy using 3D printed patient‐specific tissue compensators. He completed his Ph.D. in Medical Physics in May, 2018, and also holds an undergraduate degree in Physics from Brigham Young University.

## 2 OPENING STATEMENTS

### 2.A Eric Ehler, PhD

Phantoms provide medical physicists a means to assess the performance of medical devices in imaging, nuclear medicine, and radiation therapy.^1^ Historically, phantoms were designed and constructed by clinical staff and/or hospital engineers using materials and formulations available to them at the time.^2^ Currently, many vendors in the medical physics market provide a wide array of phantoms for clinical use. The reason for this shift could reasonably be attributed to convenience and in the interest of standardization of quality check (QC) procedures and quality assurance (QA) programs.

3D printing has been around since 1980s.^3^, ^4^ The expiration of patents related to 3D printing has lowered the cost of 3D printers. 3D printing technology has been described as the democratization of manufacturing; 3D printing is shifting the means of manufacture from a centralized system to a distributed network. The impact of increased access to manufacturing capability will reduce the convenience factor of commercial phantoms as clinicians can custom design and print phantoms as needed.

The argument “3D printing technology will eventually eliminate the need of purchasing commercial phantoms for clinical medical physics QA procedures” is already becoming reality. In most clinics, the Linac morning QA is performed with a commercial image guidance radiotherapy (IGRT) phantom, which is a cubic phantom with marks on the faces for laser alignment and embedded features for x‐ray imaging. An IGRT phantom with submillimeter accuracy was fabricated and reported by Woods et al.^5^ using computer‐aided design freeware and a relatively low cost 3D printer (commercially available for $3150 USD). In our clinic, rather than purchasing multiple identical IGRT phantoms, our team designed our own phantom in a similar manner as Woods et al. The phantom was 3D printed with PET‐G plastic for a cost of $10, using a 3D printer in a cost range of $900. The 3D printed phantom did not have the full capabilities of our commercial IGRT phantom but it fits our clinical needs as we did not fully use the features of the commercial phantom during morning QA. Additionally, when compared to a commercial small animal PET/CT imaging phantom, the 3D printed phantom was described as “functionally equivalent to commercially available phantoms”.^6^ 3D printed phantoms have also been described for MRI^7^ and PET/MRI^6^ systems. A feature of these phantoms is that they can be customized and produced by the end users at a low cost.

3D printed phantoms have been explored as patient specific phantoms for use in intensity modulated radiotherapy (IMRT) QA,^8^, ^9^ vascular imaging,^10^ and molecular imaging.^11^ For IMRT QA, 3D printing a patient specific phantom for every patient treated with IMRT is not currently clinically feasible, mostly due to time constraints. However, for commissioning new procedures or for a periodic QA schedule, using a 3D printed phantom is warranted. The use of patient specific phantoms allows for a true end‐to‐end test on a per‐patient basis at reduced cost of commercial, nonpatient specific, anthropomorphic phantoms.

Beyond phantoms, 3D printing has been investigated for radiation therapy immobilization devices,^12^ bolus,^13^, ^14^, ^15^, ^16^ electron blocks,^17^ and other treatment aids. In fact, the strongest argument for clinical acquisition of 3D printing technology is for the fabrication of treatment devices due to the unique nature of patient anatomy and the high frequency of use of treatment devices. If clinics possess 3D printers for the purpose of treatment device fabrication, the convenience of 3D printing phantoms will increase greatly.

A word of caution: 3D printing materials are not tightly controlled by all 3D printing material suppliers. For example, slight differences in formulation of 3D printing materials may affect the radiographic or other physical properties of the material. This variation could arise between one material supplier and another or even from batch to batch of the same supplier. Also, 3D printers can have defects in the printed object such as small unintended air voids or warping during printing. Air voids can occur from imperfect material deposition during the printing. Warping is an issue for fused deposition modeling (FDM) where a plastic filament is melted, extruded out of a nozzle, deposited, and then cools. Cooling can cause contraction, which may cause the FDM 3D printed object to warp. For charged particle radiation beams especially, this can negatively impact the performance of the 3D printed device or phantom.^18^ Therefore, QC of the manufacturing process will need to be performed by 3D printing staff or clinicians whereas for commercial phantoms, QC is performed by the vendor and verified by the clinicians. For example commercial water equivalent plastic blocks are usually supplied with a certificate stating the physical dimensional accuracy of the plastic, uniformity of the plastic, and the attenuation properties of the plastic. If the blocks are 3D printed by clinic staff, these tests will need to be performed in‐house.

In summary, I believe there is already a market advantage for the clinical use of 3D printed phantoms. As 3D printers gain use in routine clinical device fabrication, their utilization in other clinical areas, such as phantom fabrication, will expand. In the long term, as 3D printing capabilities increase and 3D printing materials are designed specifically for medical physics use, 3D printed phantoms will increasingly replace commercial phantoms for clinical QA procedures.

### 2.B Daniel Craft, PhD

3D printing is a transformative technology that allows users to physically manufacture anything that they can model with a computer. Over the last several years there has been enthusiastic and rapid adoption of 3D printing technology in medical physics to create a wide spectrum of custom, patient‐specific devices. 3D printers are well‐suited to manufacture a number of devices that are currently much more expensive, or much more inconvenient to procure from commercial vendors. These include customized, patient‐specific bolus and customized phantoms that may only be used once, or for a single patient. However, despite the interesting applications and enormous potential of 3D printing technology for some radiotherapy applications, presently, there are several limitations that will prevent it from being uniformly adopted as the preferred phantom fabrication technique in hospitals across the country.

The first major limitation of 3D printing is the material properties of 3D printed parts. 3D printable materials must have some specific properties; they have to either be a thermoplastic with a glass transition temperature near 200°C, or a photopolymerizing resin. This effectively limits the number of potential materials to thermoplastics and things that can be mixed with them. If a material cannot be melted and turned into a filament, it generally cannot be 3D printed. There are some creative materials that mix in other substances — like wood shavings or copper powder — with thermoplastic bases to create materials with slightly different densities and HU values, but these material differences are mostly cosmetic and intended for hobbyist 3D printing. Importantly, there currently are no commercially available materials that can replicate either bone or lung tissues. Most current 3D printed phantoms either ignore bone entirely^8^, ^19^ or use custom in‐house mixed materials to mimic bone that requires custom filament creating equipment.^20^ The first solution reduces the usefulness of the phantom, and the second solution dramatically reduces the convenience that 3D printing was supposed to provide in the first place. Similarly, the lungs are usually left open, or printed with “low infill” that matches lung density but is highly variable depending on the direction of an incident radiation beam.^21^, ^22^ Contrast this 3D printed phantom with a common commercial anthropomorphic phantom which comes with several different tissue types, including bone, cartilage, brain, soft tissue, and lung (Computerized Imaging Reference Systems, Inc. A Castleray company, Norfolk, VA). Additionally, these phantoms’ low density material properties do not depend on the direction of incident radiation like low density 3D printed phantoms.

Even if a full range of perfectly matched 3D printable materials were to be found, there are still large variations between identical 3D printed parts. We have previously shown that identically printed blocks of material can vary in density from each other up to 7%,^23^ and that is using the same printer, the same model, and the same roll of filament. There are currently dozens of different kinds of 3D printers in use in clinics around the country using many different materials and printer settings. If 3D printing QA devices becomes commonplace, it will be difficult to make meaningful comparisons of measurements across institutions that are using different 3D printers to produce phantoms based on their own specific materials and printing protocols.

Another problem with wide adoption of 3D printing is increased cost and complexity. To be clear, the actual material costs to 3D print a simple phantom are almost certainly less than the cost to purchase a similar commercial phantom. The cost of 3D printers, however, can range anywhere from several hundred dollars to several hundred thousand dollars, with a commensurately huge range in printer complexity, print quality, available features, material compatibility, and reliability. For example, the cheapest 3D printers available on Amazon.com cost less than $200, but can only print using PLA filament, have minimum layer resolutions of approximately 200 microns, and have a build volume of only a few centimeters in any direction. On the other end of the spectrum, the HP Jet Fusion 3D 3200 uses multi‐jet fusion technology to dynamically blend plastics to create parts up to 30 cm in each dimension with multiple colors and material properties, and has a minimum layer resolution of 70 microns. However, its cost starts at $155,000. It is important to remember that in‐house phantom production will require in‐house 3D printing expertise, so will it be the medical physicist's responsibility to be proficient in 3D design as well as the mechanical operation and maintenance of a 3D printer? Whose responsibility will it be if the 3D printer jams during a print and patient QA cannot be performed? 3D printers mostly operate in the background, but they do require operators to plan and start models printing, as well as change out materials and occasionally replace parts. Especially with less expensive printers the user must be able to troubleshoot and fix errors. This may be feasible in larger academic centers, but I do not think it is a reasonable expectation that the many small or nonacademic clinics that make up the majority of cancer care will embrace this unnecessary increased workload.

In conclusion, 3D printing is currently not a mature enough technology to become the primary technique for fabricating important QA devices in radiotherapy clinics. Conventionally fabricated commercial phantoms are more uniform, reliable, and simple than 3D printed ones. It is definitely true that 3D printing has a place in radiation oncology — and an exciting one at that! The question that 3D printing must address is: what additional value does it provide over conventional phantom fabrication that outweighs the previously mentioned limitations. In my opinion, that value is in creating highly customized or unique phantoms for research and development in major academic centers, not in creating routine QA phantoms that every clinic needs. I am confident that 3D printing will eventually replace *some* commercial phantoms for clinical medical physics QA procedures at *some* clinics, but definitely not for all, or even most of them.

## 3 REBUTTAL

### 3.A Eric Ehler, PhD

I agree with Dr. Craft that currently there are many difficulties to overcome. However, in the long‐term view I maintain the argument that all QA phantoms will be fabricated with 3D printing.

It is true that currently available 3D printing materials are not equivalent to human tissues. Attributable to the complexity in designing a material that is compatible with 3D printing and is tissue or water equivalent, materials science developments are needed. In the meantime, there is an alternative to fully 3D printing a phantom if it is desired to be tissue or water equivalent. That is to use 3D printing to create a mold to fill with an equivalent material(s); this strategy can be used for phantoms^9^ as well as radiotherapy bolus.^15^ This can reduce 3D printing times and bypass deficiencies in the radiologic properties of current 3D printing materials such as those demonstrated by Dr. Craft.^23^

Regarding 3D printer QA and additional workload, monitoring printers for jams or other print failures can be performed with a software packages such as OctoPrint. The software can be used to monitor printing progress via webcam and, if necessary, the print job can be aborted remotely. Updates on the printing progress can even be sent to mobile devices. To lend perspective on the frequency of print failures, one of our printers (Lulzbot Taz 6) has over 200 print hours with only one failed part in that time while a previously used printer failed quite regularly; thus the choice in the 3D printer is important. In addition, it is true that QA will be required for 3D printed phantoms or devices. However as physicists, we are responsible for the materials and devices used clinically. Regardless of whether a phantom is fabricated in‐house or purchased from an established vendor, validation of the phantom and implementation into clinical use is required. There may be additional considerations in the QA of 3D printed phantoms or devices, but the advantages offset the additional workload.

Finally, I contest the statement that 3D printing may be feasible for large academic centers but not for smaller clinics. In fact, I believe that the greatest benefit will be to smaller clinics. At a large academic center, there are likely engineers within the hospital and engineering machine shops nearby to fabricate phantoms and devices. Smaller clinics likely lack these resources and 3D printing can fill that gap at a reasonable cost.

### 3.B Daniel Craft, PhD

There are several points upon which Dr. Ehler and I agree. First, and most importantly, we share a concern for some of the variable material properties that 3D printed objects can have. As he notes, different material suppliers are not held to strict material standards, which can lead to various imperfections and inconsistencies in 3D printed parts. Objects printed from different suppliers using an equivalently labeled material could have different densities and radiological properties.^23^, ^24^ This is, however, not the only potential source of uncertainty. I would add that the quality of a printed object will depend equally as largely on the 3D printer used, and the model that has been designed. There are many 3D printers with slightly different properties that could affect print quality, such as how stable it can maintain the nozzle and bed temperature, how fast the extruder moves, and many more. Additionally, unless 3D models of useful phantoms are shared across all institutions there will be additional variation between clinics in the actual characteristics of phantoms used for QA.

This leads to the second point on which we have common ground: if phantoms are printed in house, calibration and standardization tests into dimensional accuracy, material uniformity, and material attenuation properties will also have to be performed in house. As Dr. Ehler notes, these certifications currently come with phantoms from commercial suppliers. While larger research institutions may have additional resources and time to make this in house testing feasible, having to perform these tests for every printed object is an unnecessary workload for most smaller clinics. This increased workload for physicists in designing objects to be printed, maintaining a 3D printer, and validating 3D printed objects is in my opinion a major limiting factor in the widespread adoption of clinical 3D printing.

As Dr. Ehler has mentioned, another use for 3D printing aside from creating clinical phantoms is the creation of patient‐specific treatment devices. This is a very interesting application of 3D printing, because many of these devices are currently difficult, time‐consuming, or expensive to acquire through conventional fabrication. With 3D printing, however, patient specific bolus^13^, ^15^, ^25^ can be rapidly and inexpensively produced that reduces air gaps and improves dosimetric plan characteristics relative to less conformal bolus. In fact, I agree with Dr. Ehler that “the strongest argument for clinical acquisition of 3D printing technology is for the fabrication of treatment devices.” I disagree, however, with his assertion that this technology can be applied equally to creating phantoms for every clinical need. Although 3D printed bolus is in many ways more convenient than and superior to conventional bolus, 3D printed phantoms are generally harder to manufacture and have inferior material properties relative to conventional phantoms.

Ultimately, the debate around 3D printing taking over conventional commercial phantoms is an argument of magnitude. It is clear that 3D printing is currently being used in clinics around the country for a variety of interesting purposes including phantom development,^9^, ^11^ treatment device fabrication,^13^, ^16^ and more.^7^, ^26^, ^27^ As the technology matures and continues to develop I am sure that it will improve and more use cases will be found. However, it is my opinion that 3D printing will remain a supplemental technology to fabricate a few special things, and will not ever completely replace conventionally fabricated commercial phantoms.
